# Patient Adherence to Tuberculosis Treatment: A Systematic Review of Qualitative Research

**DOI:** 10.1371/journal.pmed.0040238

**Published:** 2007-07-24

**Authors:** Salla A Munro, Simon A Lewin, Helen J Smith, Mark E Engel, Atle Fretheim, Jimmy Volmink

**Affiliations:** 1 South African Cochrane Centre, Medical Research Council of South Africa, Cape Town, South Africa; 2 Primary Health Care Directorate, University of Cape Town, Cape Town, South Africa; 3 Health Systems Research Unit, Medical Research Council of South Africa, Cape Town, South Africa; 4 Department of Public Health and Policy, London School of Hygiene and Tropical Medicine, London, United Kingdom; 5 International Health Group, Liverpool School of Tropical Medicine, Liverpool, United Kingdom; 6 Department of Medicine, University of Cape Town, Cape Town, South Africa; 7 Norwegian Knowledge Centre for the Health Services, Oslo, Norway; 8 University of Stellenbosch, Faculty of Health Sciences, Cape Town, South Africa; Michigan State University, United States of America

## Abstract

**Background:**

Tuberculosis (TB) is a major contributor to the global burden of disease and has received considerable attention in recent years, particularly in low- and middle-income countries where it is closely associated with HIV/AIDS. Poor adherence to treatment is common despite various interventions aimed at improving treatment completion. Lack of a comprehensive and holistic understanding of barriers to and facilitators of, treatment adherence is currently a major obstacle to finding effective solutions. The aim of this systematic review of qualitative studies was to understand the factors considered important by patients, caregivers and health care providers in contributing to TB medication adherence.

**Methods and Findings:**

We searched 19 electronic databases (1966–February 2005) for qualitative studies on patients', caregivers', or health care providers' perceptions of adherence to preventive or curative TB treatment with the free text terms “Tuberculosis AND (adherence OR compliance OR concordance)”. We supplemented our search with citation searches and by consulting experts. For included studies, study quality was assessed using a predetermined checklist and data were extracted independently onto a standard form. We then followed Noblit and Hare's method of meta-ethnography to synthesize the findings, using both reciprocal translation and line-of-argument synthesis. We screened 7,814 citations and selected 44 articles that met the prespecified inclusion criteria. The synthesis offers an overview of qualitative evidence derived from these multiple international studies. We identified eight major themes across the studies: organisation of treatment and care; interpretations of illness and wellness; the financial burden of treatment; knowledge, attitudes, and beliefs about treatment; law and immigration; personal characteristics and adherence behaviour; side effects; and family, community, and household support. Our interpretation of the themes across all studies produced a line-of-argument synthesis describing how four major factors interact to affect adherence to TB treatment: structural factors, including poverty and gender discrimination; the social context; health service factors; and personal factors. The findings of this study are limited by the quality and foci of the included studies.

**Conclusions:**

Adherence to the long course of TB treatment is a complex, dynamic phenomenon with a wide range of factors impacting on treatment-taking behaviour. Patients' adherence to their medication regimens was influenced by the interaction of a number of these factors. The findings of our review could help inform the development of patient-centred interventions and of interventions to address structural barriers to treatment adherence.

## Abstract 2

From a systematic review of qualitative research, Munro and coauthors found that a range of interacting factors can lead to patients deciding not to complete their course of tuberculosis treatment.

## Editors' Summary

**Background.:**

Every year nearly nine million people develop tuberculosis—a contagious infection, usually of the lungs—and about two million people die from the disease. Tuberculosis is caused by Mycobacterium tuberculosis, bacteria that are spread in airborne droplets when people with active tuberculosis sneeze or cough. Tuberculosis can be cured by taking several strong antibiotics daily for at least six months but many patients fail to complete this treatment because the drugs have unpleasant side-effects and the treatment is complicated. In addition, people often feel better soon after starting treatment so they stop taking their tablets before all the bacteria in their body are dead. Poor treatment adherence (poor compliance) means that people remain infectious for longer and are more likely to relapse and die. It also contributes to the emergence of drug-resistant tuberculosis. To help people complete their treatment, the World Health Organization recommends a strategy known as DOTS (directly observed treatment, short course). As part of this strategy, a health worker or a tuberculosis treatment supporter—a person nominated by the health worker and the patient—watches the patient take his/her antibiotics.

**Why Was This Study Done?:**

Although DOTS has contributed to improved tuberculosis control, better patient compliance is needed to halt the global tuberculosis epidemic. Treatment adherence is a complex behavioral issue and improving treatment outcomes for tuberculosis (and for other diseases) requires a full understanding of the factors that prevent people taking medicines correctly and those that help them complete their treatment. In this study, the researchers have done a systematic review (a study in which the medical literature is surveyed and appraised using defined methods to reach a consensus view on a specific question) of qualitative studies that asked patients, carers, and health workers which factors contributed to adherence to tuberculosis treatment. Qualitative studies collect non-quantitative data so, for example, a qualitative study on tuberculosis treatment might ask people how the treatment made them feel whereas a quantitative study might count bacteria in patient samples.

**What Did the Researchers Do and Find?:**

The researchers searched electronic databases and reference lists for qualitative studies on adherence to tuberculosis treatments and also consulted experts on tuberculosis treatment. They carefully read the 44 published papers that met their predefined inclusion criteria and then used a method called “meta-ethnography” to compare the factors (themes) associated with good or bad adherence in the different studies and to synthesize (reach) a consensus view of which factors influence adherence to tuberculosis treatment. The researchers identified eight major factors associated with adherence to treatment. These included: health service factors such as the organization of treatment and care; social context (family, community and household influences); and the financial burden of treatment. Finally, the researchers interpreted the themes that emerged from the studies to build a simple model that proposes that adherence to tuberculosis treatment is influenced by four interacting sets of factors—structural factors (including poverty and gender discrimination), social context factors, health service factors, and personal factors (including attitudes towards treatment and illness).

**What Do These Findings Mean?:**

The findings of this systematic review of qualitative research on patient adherence to tuberculosis treatment are inevitably limited by the quality and scope of the original research. Consequently, further studies into patients' understanding of tuberculosis and its treatment are needed. Nevertheless, the findings and the model proposed by the researchers indicate that patients often take their tuberculosis medications under very difficult conditions and that they cannot control many of the factors that prevent them taking their drugs. So, although current efforts to improve adherence to tuberculosis treatments emphasize instilling a willingness to take their medications into patients, this systematic review suggests that more must be done to address how factors such as poverty and gender affect treatment adherence and to tailor support systems to patients' needs. Most importantly, it indicates that future interventions should involve patients more in the decisions made about their treatment.

**Additional Information.:**

Please access these Web sites via the online version of this summary at http://dx.doi.org/10.1371/journal.pmed.0040238.

- MedlinePlus has an encyclopedia page on tuberculosis (in English and Spanish)
- See the US National Institute of Allergy and Infectious Disease fact sheet on tuberculosis
- US Centers for Disease Control and Prevention provide a variety of fact sheets and other information resources on tuberculosis
- World Health Organization has produced the 2007 Global Tuberculosis Control report (in English with key findings in French and Spanish), information on DOTS (in English, Spanish, French, Russian, Arabic and Chinese), and A Guide for Tuberculosis Treatment Supporters
- See the brief guide to systematic reviews, published by the *British Medical Journal*

## Introduction

Tuberculosis (TB) is a global health concern, with an estimated 8.9 million new cases worldwide in 2004 and two million deaths each year [1]. It is a major contributor to the burden of disease, especially in low- and middle-income countries, where it is being fuelled by the HIV/AIDS epidemic [2].

DOTS (directly observed treatment, short course) is the internationally recommended control strategy for TB [3]. This strategy includes the delivery of a standard short course of drugs, lasting 6 mo for new patients and 8 mo for retreatment patients, to individuals diagnosed with TB. The delivery includes the direct observation of therapy (DOT), either by a health worker or by someone nominated by the health worker and the patient for this purpose (sometimes called a DOT supporter). The strategy has been promoted widely and implemented globally.

Up to half of all of patients with TB do not complete treatment [4], which contributes to prolonged infectiousness, drug resistance, relapse, and death [5]. The difficulty experienced by patients following a particular treatment regimen has raised awareness of adherence as a complex behavioural issue, influenced by many factors [6], including gender and the impact of HIV/AIDS. WHO has attempted to classify factors that influence adherence to TB treatment based on a cursory review of key papers [6], but the impact of gender [7] and HIV status [8] on adherence are less well documented in the qualitative literature.

Efforts to improve treatment outcomes require a better understanding of the particular barriers to and facilitators of adherence to TB treatment, and of patient experiences of taking treatment [9]. Qualitative research can contribute to this understanding and help interpret the findings of quantitative studies of the effectiveness of adherence-promoting interventions [10]. The volume of such qualitative research is growing and we believe that one way to draw useful lessons from this literature is by synthesising the findings of these studies.

Systematic synthesis of relevant qualitative studies of TB treatment adherence can provide more complete knowledge than that derived from individual studies alone. It can assist in the interpretation of findings of single studies; help explain variation or conflicts in study findings; enable the development of new theories; and help inform the design of new interventions. In addition, it may allow the identification of gaps in existing adherence research.

In this review we consider the perspectives of patients, caregivers, and health care providers regarding adherence to TB treatment. The findings of this review will have implications for a range of stakeholders including nongovernment organisations, national policy makers, and international bodies working towards reducing the global health burden of TB.

## Methods

**Figure 1:**
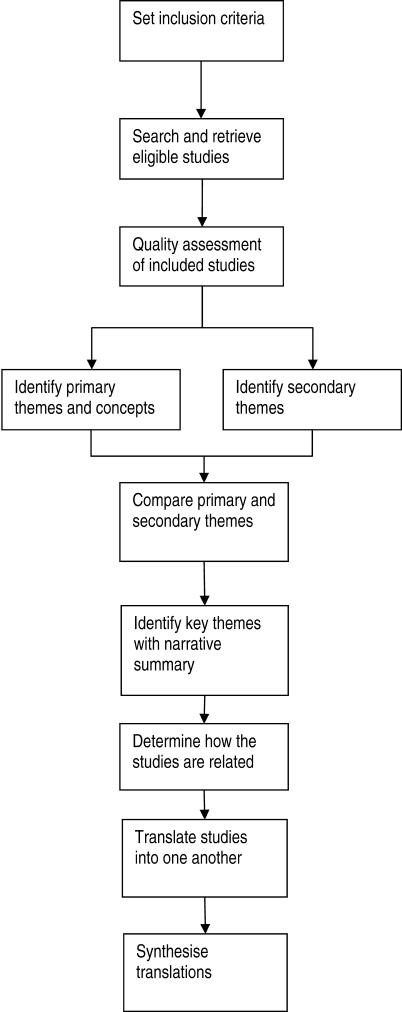
Meta-ethnography Process

We followed a meta-ethnographic approach [11], the steps of which are outlined in Figure 1, to synthesise findings across included studies. This systematic approach translates ideas, concepts, and metaphors across different studies and is increasingly seen as a favourable approach to synthesising qualitative health research [11,12]. The research team included three social scientists (SM, SL, HS) and three clinical researchers (JV, AF, ME). The social scientists had different disciplinary backgrounds.

### Inclusion Criteria

We included studies that examined adherence or nonadherence to preventive or curative TB treatments and described the perspectives of patients, care givers, or health care providers. We included studies from any discipline or theoretical tradition that used qualitative methods. We included papers that reported qualitative research only, as well as research using qualitative and quantitative methods (mixed method) that reported qualitative findings. Both published and unpublished studies reported in English were considered. Because of resource limitations, papers published in other languages were not considered.

### Search Strategy and Study Selection

**Figure 2:**
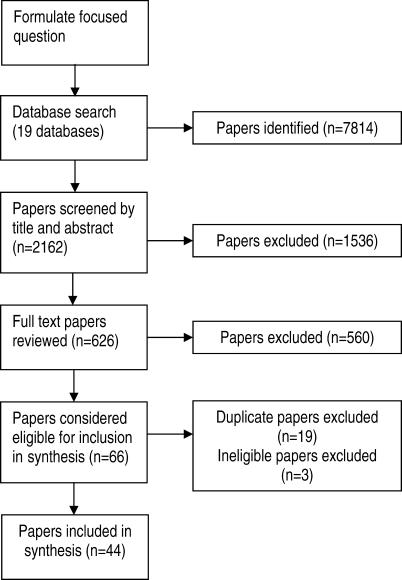
Search Process and Study Selection

Figure 2 maps out the process by which articles were selected for our systematic review. We searched 19 databases, using the keywords: “TB AND (adherence OR concordance OR compliance)” from 1966, where available, until 16 February 2005 (see Table S1 for search results). This process was complemented by reviewing citations, searching in Google Scholar, and expert referrals. Additional articles were included as they became available. We used the search, assessment, and retrieval process outlined by Barroso et al. [13]. SM scanned more than 7,000 citations identified in the various databases and retrieved abstracts for potentially relevant studies (*n* = 2,162). Approximately 10% (*n* = 222) of these were also reviewed by JV to validate the selection of articles. Disagreements (*n* = 17 papers) were resolved by discussion and reference to the full article. Thereafter, SM screened the titles and abstracts of potentially relevant studies, excluding 1,536 papers and retrieving potentially eligible papers (*n* = 626). After scanning the full text, 560 of these articles were not considered eligible and 66 were considered potentially eligible, based on our inclusion criteria. The abstracts of these were assessed by SM and SL, and ineligible and duplicate papers were excluded, leaving 47 that were considered eligible. Two independent reviewers then read the full paper of each study, following which three more papers were excluded because they did not include qualitative data or because they had insufficient descriptions of data collection or analysis methods. The final synthesis therefore involved 44 papers.

### Quality Assessment

We decided to assess the quality of individual studies using a checklist based on common elements from existing criteria for qualitative study quality assessment [10,14–17] (Table 1). These existing checklists are published and peer reviewed, but unlikely to be validated; only the Critical Appraisal Skills Programme criteria [17] have been used by other meta-ethnographers [18]. Evaluating study quality allowed us to describe the range of quality across included studies. Two reviewers independently assessed study quality using a pretested form and resolved differences by discussion. No studies were excluded on the basis of quality. This approach was taken for two reasons: first, both the original authors of the meta-ethnographic approach [11], and other users of the method [19], have found that poorer-quality studies tend to contribute less to the synthesis. The synthesis therefore becomes “weighted” towards the findings of the better-quality studies. Second, there is currently no consensus among qualitative researchers on the role of quality criteria and how they should be applied [10], and there is ongoing debate about how study quality should be assessed for the purposes of systematic reviews [20].

**Table 1:**
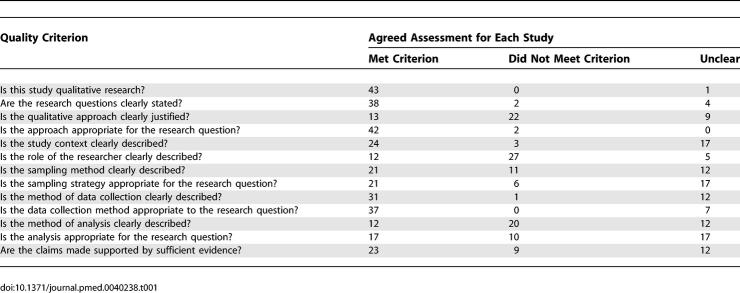
Methodological Quality of Included Studies (*n* = 44)

### Synthesis

Based on the meta-ethnography approach described by Noblit and Hare [11], we used reciprocal translation, analogous to constant comparison in primary qualitative research, to compare the themes identified in each study. We then conducted a “line-of-argument synthesis,” an approach similar to grounded theory in primary research, to determine a model of factors influencing treatment adherence. From this process we derived hypotheses relating to the reorganisation of treatment and care to improve adherence. The synthesis process is described below and illustrated in Figure 1.

#### Identifying themes and concepts.

We identified concepts, themes, and patterns by reading and rereading the included studies. In this process, we understood primary themes or first-order constructs as reflecting participants' understandings, as reported in the included studies (usually found in the results section of an article). Secondary themes or second-order constructs were understood as interpretations of participants' understandings made by authors of these studies (and usually found in the discussion and conclusion section of an article). However, we recognise that all reported data are the product of author interpretation [21]. One author (SM) extracted first- and second-order constructs from the articles, plus relevant data on study context, participants, treatment type, and methods using a standard form. The rest of the study team independently extracted data from half of the studies, but found no major differences. Although the foci of the studies were not all directly comparable, the study team identified a number of recurring first- and second-order constructs.

#### Determining how the studies are related.

We used thematic analysis to inductively develop categories from the first-order themes and concepts identified in the included studies. These categories represent related themes and concepts and initially included: family, community, and social support; professional practice and organisation of care; financial burden; personal characteristics as related to treatment adherence; access to services; disease progression; and knowledge, beliefs and attitudes towards treatment. We revised and merged these categories by discussing together as a team how they were related. We followed a similar process for second-order constructs identified from the included studies.

#### Reciprocal translation of studies.

Following the meta-ethnographic method closely, we compared the concepts and themes in one article with the concepts and themes in others. Translation involves the comparison of themes across papers and an attempt to “match” themes from one paper with themes from another, ensuring that a key theme captures similar themes from different papers (see Britten, et al. for further description [12]). We approached the reciprocal translation by arranging each paper chronologically, then comparing the themes and concepts from paper 1 with paper 2, and the synthesis of these two papers with paper 3, and so on. We began with the categories identified in the process described above, but incorporated others as they emerged. Two authors conducted the translation independently, returning to the full-text papers frequently throughout. In this review our aim was to explore adherence to TB treatment without confining this variable to a specific population or subgroup, but in doing so we were careful not to inappropriately synthesize the findings of heterogenous studies. In the process of comparing the studies against each other, we looked for explicit differences between the studies in relation to a range of factors including their geographic location, socioeconomic conditions, and the type of treatment programme.

From the reciprocal translation we were able to construct tables showing each theme and related subthemes, and narratives to explain each theme.

#### Synthesising translations.

**Figure 3:**
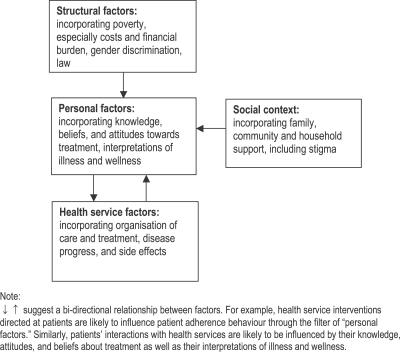
Model of Factors Affecting Adherence

We chose to synthesise the results of the translation independently to account for different interpretations by disciplinary background. To develop an overarching framework (or third-order interpretation), we listed our translated themes and subthemes in a table, juxtaposed with secondary themes derived from author interpretations (see Table 2). Each member of the research team then independently developed an overarching framework by considering if and how the translations and authors' interpretations linked together. From this we produced a model (Figure 3) and generated hypotheses, in a “line-of-argument” synthesis. Line-of-argument syntheses create new models, theories, or understanding rather than a description of the synthesised papers [11].

**Table 2:**
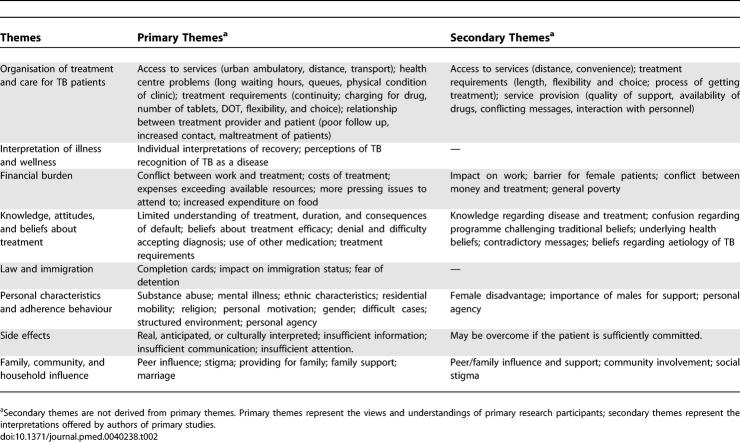
Primary and Secondary Themes Emerging from the Included Studies

We attempted to explore systematically the influence of socioeconomic status and geographic location on the findings of our synthesis. However, it was difficult to determine many patterns except those highlighted specifically by authors of the primary research. We realised that synthesising studies from a variety of contexts would present challenges, but also felt that including these studies would provide an opportunity in the synthesis to explore the differences between the contexts, if these existed. Similarly, we chose to include studies examining adherence to latent TB treatment as well as adherence by injecting drug users (IDUs) and homeless people, with specific attention being paid to the ways that the issues raised in these studies differed from those focused on active TB in other populations. Again, few differences emerged.

## Results

### Description of Studies

Forty-four studies published between 1969 and 2006 were included in the review. The studies were conducted in Africa (14), North America (9), South (8) and East Asia (8), Latin America (2), and Europe (2). It was difficult to discern the study setting from the published reports, but most were conducted within a clinic or health service setting (see Table 3). Most studies were concerned with curative TB treatment (33); others focused on preventive treatment (8) and some considered both (2). Most of the studies involved TB patients, often also including community members and health care workers. Three studies involved IDUs and homeless individuals. Approximately 3,213 individuals were involved in the included studies. We found few studies that justified their use of a qualitative approach (*n* = 13) or specified the underlying theoretical framework (*n* = 10), and few authors reported on their role as researcher (*n* = 12) (Table 1). In 12 papers the method of analysis was clearly described, but some derivation of thematic analysis appeared to be used in others. Although several studies seemed to have high face validity, they often scored poorly on our quality assessment instrument, possibly due to the instrument's ability to measure only the quality of reporting.

**Table 3:**
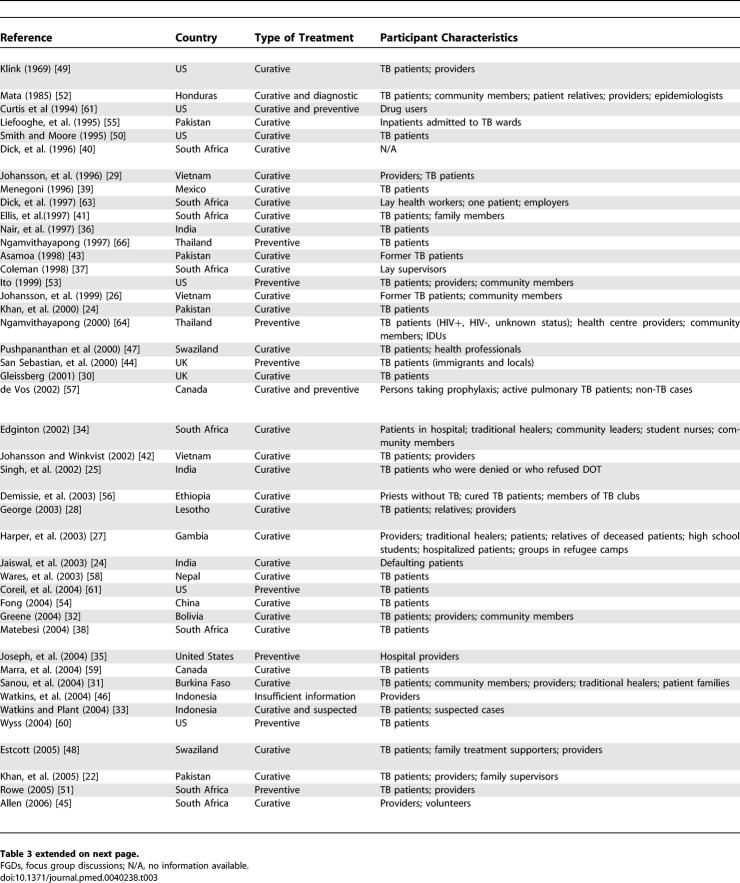
Characteristics of Primary Studies Included in this Review

**Table 3:**
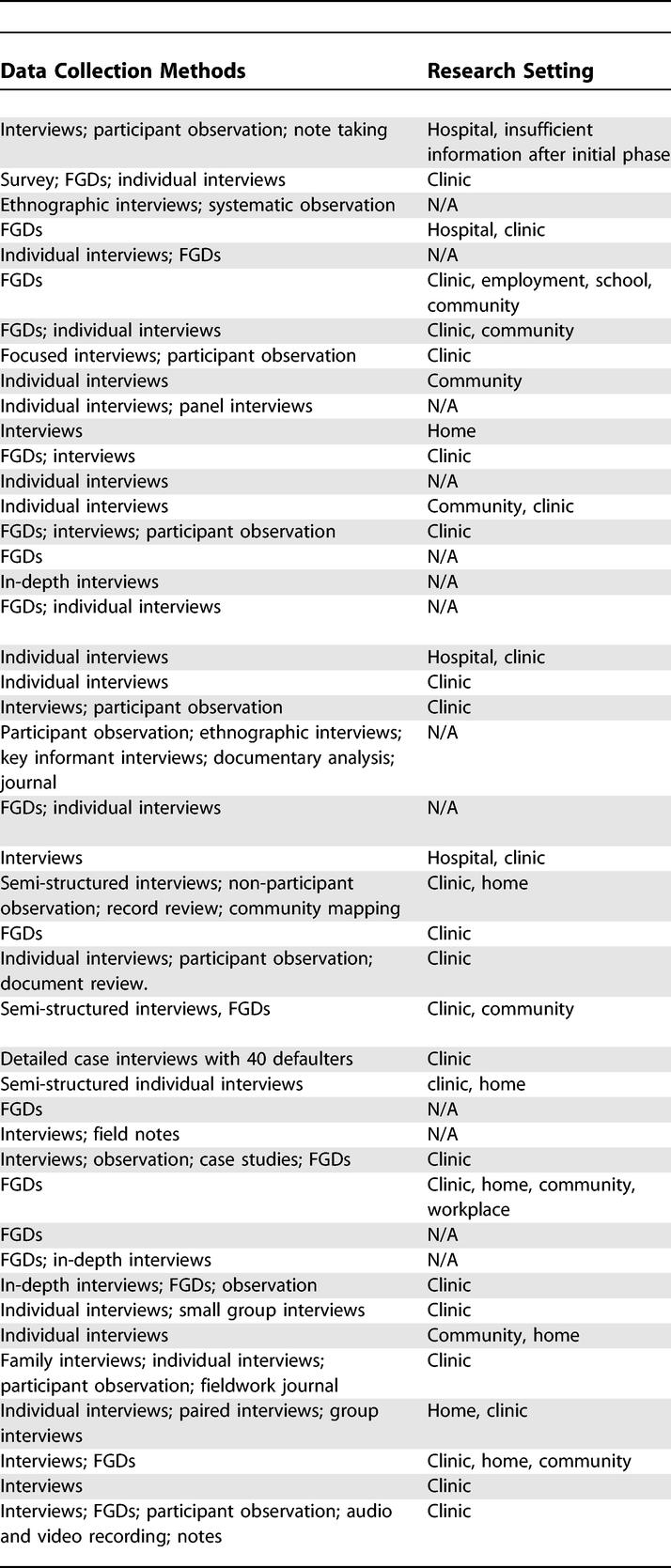
Extended.

### Description of Themes

Eight primary themes (identified from participants' understandings) and six secondary themes (derived from authors' interpretations) arose from the synthesis (Table 2). Each primary theme is described in Boxes 1–8 using direct quotes to illustrate meaning.

**Box 1: Organisation of Treatment and Care for TB Patients**

“The patients do not have the adequate means to go to the health centre to take their drugs. They just have camel, donkey or carts… And sometimes, the state of some patients prevents them from using these” (male family member of TB patient, Burkina Faso) [31].

“A dirty place can affect the psychology. It makes people lose heart and feel unenthusiastic about continuing treatment” (female participant with TB, Vietnam) [26].

“It just does not make sense as to why a grown up person should be given medicines by someone else. I felt very awkward, and tried to take my medicines myself” (male TB patient, Pakistan [22].

“…and I was afraid to go to the doctor, I thought he would scold me because I missed treatment for a day. For this reason, I didn't go back to get more pills. I was afraid…” (female participant, Bolivia) [32].

“The minute you tell them you're homeless they treat you real snobbish… They treat you like a dog down there once you get past the triage nurse…” (female TB patient, United States) [50].

‘…It did help, cos I really needed assurance that it was definitely going to be [cured] and doctor spent a lot of time with me. And they were really, really um, they were outstanding there” (male TB patient, United Kingdom) [30].

**Box 2: Interpretations of Illness and Wellness**

“…When I feel better, I don't take the tablets. Only when I feel pain” (completer, South Africa) [51].

“…She said ‘no no no I do not have TB any more' because she no longer has blood in her sputum” (provider, Indonesia) [46].

“Well, if you know a little bit about the disease and, like we say, if it's latent… you are not sick. It's only.. if it becomes active, then you are liable to be sick and probably very sick. So then you consider taking the medicine that is terribly bad: which is worse? That's when you weigh what is best for you” (provider, United States) [35].

“I think that I feel healthy, my lungs are good, but I have a bit of fear that the sickness will return… But as I told you, I don't want to take these pills, because they make me sick, they hurt me…. “ (female TB patient, Bolivia) [32].

**Box 3: Financial Burden of TB Treatment**

“It's a bit difficult, because, as I told you, the radiography and the control smear cost more than 100B; the consult costs 15B…it will cost me almost 150B to start treatment again. At this moment, I don't even have the money for the trip to the hospital...” (male TB patient, Bolivia) [32].

“TB here is closely related to social and economic problems. People live in densely populated areas, their income is poor, and they don't understand about TB” (provider, Indonesia) [46].

“We cannot remain out of a job for long. As soon as we feel better we would like to go to work… If I cannot earn, my whole family will suffer” (male TB patient, South Africa) [51].

“Typically it [treatment] would be three months.. that's a long time for anyone to be available without any compensation… it's tremendously a matter of economics and economics only…” (male TB patient, Canada) [57].

**Box 4: Knowledge, Attitudes, and Beliefs about TB Treatment**

“He believed that he should always use the expensive tablets and not the tablets from [the health care facility]. The … tablets were not correct with the problem inside, and the colour of the tablets doesn't look right” (participant, Indonesia) [33].

“No doctor is able to cure this” (patient, South Africa) [34].

“That's just like basic common sense, this is no test… if the doctor says to us take these tablets then that's common sense.” (male TB patient, UK) [30].

“…And when you take medications, these bugs will die, he told me. The medications kill the bugs. This is what I've been told, but I'm not sure. It seems uncertain to me. Because the pills didn't help me….” (female TB patient, Bolivia) [32].

“…a lot of people don't take the medicine because they feel that taking it doesn't do any good for their health” (female noncompliant patient on prophylaxis, US) [53].

**Box 5: Law and Immigration**

“Because the nurse tells us that here they have a record of people who have TB, and when they go to apply for a job it shows up on the record that they have TB and it was untreated, they need [the completion record] for the job” (male Vietnamese refugee patient, US) [53].

**Box 6. Personal Characteristics and Adherence Behavior**

“How would somone who starts drinking early in the morning visit the clinic? Some patients consume alcohol daily. They would rather decide to interrupt their treatment, than discarding their drinking habit” (male respondent, South Africa) [40].

“…When my husband went back home, he was angry with himself and he was upset about everything. He refused to eat and rejected his medicine. He threw his pills away. He did not take TB medicine at all” (female HIV+ TB patient, Thailand) [64].

“[interviewer: ‘Some people don't want to take their pills]’ Stupid people, sorry to say that” (male TB patient, UK) [30].

“I missed taking some pills because I was drunk or high on drugs” (female TB patient, US) [59].

**Box 7: The Influence of Side Effects on Treatment Adherence**

“…Unpleasant metallic taste in his mouth… asked if a non-vegetarian diet would improve this problem. He was laughed at by the [provider] along with a number of others in the clinic and some personal remarks were made…he finally left treatment” (male TB patient, India) [24].

“I said no wonder they defaulted, many of them defaulted, you know, because it is [side effects] just too much, it is just too much …” (TB patient, UK) [30].

“These tablets let one's body itches for the whole day. I know someone who interrupted this treatment because of this problem.”(male TB patient, South Africa) [38].

“…I don't want to take these pills, because they make me sick, they hurt me…” (female TB patient, Bolivia) [32].

**Box 8: Family, Community, and Household Influences**

“I arrive early in the morning so that people could not see me. I used to conceal my illness from people… People think that we are the filthiest people… it was really difficult to accept that I have TB” (male patient, South Africa) [40].

“We are two sisters and marriage arrangements have been made with men from one family. If my (future) family-in-law knows that I have TB they will be sure then to break the engagement...I'm worried for my sister. Her engagement also could break off because of my sickness” (female patient, Pakistan) [55].

“Just pick up the medication even if you don't use it” (patient advice to another patient on preventive treatment, US) [53].

“…I must have responsibility to take care of my child… If I die, who will take care of her? …. When I think of my child… I must be cured. This made me feel I must take the medicine” (female HIV-positive TB patient, Thailand) [64].

“…It was very important, I had my sister and my ex-girlfriend and it was really, really important to have someone, you know, to give you support especially when you don't know much about the disease” (male TB patient, UK) [30].

“…Since I have three children that I need to support… this worried me more” (male TB patient, Bolivia) [32].

We found no discernible patterns when we explored the influence of factors such as geographic location, socioeconomic status, latent or active TB, type of treatment programme, or special groups such as IDUs or the homeless. Although some studies differentiated between patients receiving treatment in urban and rural areas, no strong differences emerged between these settings, and we therefore judged it appropriate to synthesize findings across all studies. Any differences that emerged between studies with regard to specific factors are noted in the text below.

### Organisation of Treatment and Care for TB Patients

For most patients, access to a health care facility depended on distance and available transport as well as their physical condition. One study indicated that, although the intention was for a DOT supporter to visit the patient's home, in practice the patient had to walk to the supporter's home [22]. This proved especially difficult for patients with severe symptoms [22–25]. One study noted that access to health care facilities was better in urban areas than rural areas [26], and both patients [27,28] and providers [29] noted that adherence was compromised if the distance from patients' homes to the nearest clinic was too great. If patients' homes were close to a clinic, however, the patients could attend regularly [30]. For patients on DOT, the time needed to present for direct observation of treatment-taking compromised their ability to attend to other daily tasks [25,31,32]. In one study, patients found private practitioners more accessible [26].

Problems manifesting specifically at health facilities included long waiting times, queues, lack of privacy, inconvenient appointment times [23,26–28,31–35], and the poor upkeep of clinics [26,27]. Many studies reported that patients experienced difficulty in accessing treatment at health care facilities because of inconvenient opening hours and provider absenteeism [22,23,31,37–38]. Poor TB medication availability at health care facilities was highlighted by patients [23,33,36,38] and providers [29]. For example, one study reported that a health care worker sold TB medication that should have been freely available [31]. A patient's relationship with the treatment provider also appeared to influence adherence. A large number of studies indicated that poor follow-up by providers [33,36,39], and maltreatment by providers [23,24,31,39–41], such as scolding a patient for missing appointments, resulted in nonadherence. In contrast, other studies noted the positive impact of increased provider–patient contact on adherence [26,39,42,43].

Some studies highlighted how treatment requirements could impact on patient attitudes towards treatment and thus on adherence behaviour. Patients could “become tired” of taking medications [26,30,40,44,45], discontinuing because of the length of treatment [38,40,45,46], the number of tablets [24], or fear of painful injections or drugs [29,47], as noted by both providers and patients.

Some patients reported they found it difficult to meet the requirements of DOT [24,25,32,39,40]. In a number of studies conducted with patients being directly observed [22,24,34,42], adherence to treatment was facilitated by flexibility and patient choice. The continuity of the treatment process was important to patients [39,42], and irregular supervision by a family member sometimes compromised the treatment programme [22,23]. Some patients viewed direct observation negatively [22–25,40,45,48], interpreting it as distrust, and in one study describing the process as “doing time” [49]. In contrast, a study conducted with IDUs indicated that these patients appreciated the direct observation component of care because they received their treatment together with their methadone from a street nurse [50].

### Interpretations of Illness and Wellness

Studies in our synthesis reported that patients stopped treatment because they felt better and thought that they were cured [23,24,39,40,45,47,49,51] or because their symptoms abated [47,52,53]. Some studies noted that patients who felt worse than before treatment [23,24,32] or saw no improvement in their condition [22–24,46] might be more likely to interrupt treatment. A study conducted in The Gambia reported that migrants arrived in the country to receive TB treatment and returned home once they felt better [27]. This problem may be linked to patients' conceptions of recovery, and of the aetiology of TB.

Treatment interruption was also reportedly related to perceptions about TB as a disease; some patients did not believe that they had TB, only wanted a cure for their symptoms and ceased treatment once these lessened [33,43,52]. Another study reported that patients were motivated to continue treatment as a consequence of symptom relief [30]. One study conducted in China noted that patients often continued to take medication after the necessary period of six months, and some patients would continue with treatment despite not having any symptoms, because they believed that the “roots” of the disease needed to be removed [54].

Some patients needed help in taking their medication when they were too weak [23], while others on preventive treatment and with no symptoms hesitated to even begin treatment, thinking that it could make them ill [35]. Three studies found that patients experiencing severe symptoms were more likely to adhere [39,43,54], possibly due to a fear of becoming more ill.

### Financial Burden of TB Treatment

Several studies indicated that having TB had consequences for work [22–24, 26,27,29,32,34,42,52,54–56]. Studies suggested that patients hide their disease for fear that employers may discover that they have TB, with consequent effects on adherence. Additional work-related issues included difficulty in obtaining sick leave for treatment; fear of asking for money to purchase TB drugs; and fear of losing work or dismissal [26,29,36,55].

The reports showed how some patients prioritised work over taking treatment—and for many there appeared to be a “choice” between work and adherence [23,24,26,29,32,34,36,37,42,45,54]. More common in rural areas, this was not a real “choice” but rather a conflict between attending for clinic-based treatment and the need to earn a living. This was manifested in patients feeling “forced” to choose between work and attending treatment [26]; patients having “no choice” but to abandon treatment because it was too difficult to combine the two [29]; and patients not being able to afford treatment, but if they sought work, being unable to attend for treatment [32]. A study with inner-city homeless people on preventive treatment reported that treatment posed an economic barrier for them because they often worked out of town [57]. Patients also expressed guilt over the impact that the disease had on their family livelihoods [31]. Several studies found that patients had more pressing issues to attend to in everyday life [24,29,31,32,40,42,45,56], such as taking care of family. Economic constraints were especially noted in rural areas, especially for patients on preventive treatment [51].

Patients often explained treatment interruption by noting the costs of treatment [23,26,29,32,33]. In some settings, patients reported that drugs were expensive [29,36] and, where treatment itself was free, hidden costs such as hospital stays [29], reviews of X-ray results, and transport costs could be high. In some cases providers acknowledged patients' financial constraints [31]. However, there were examples of doctors not accepting that costs caused patients to stop taking treatment because, from the doctors' perspective, treatment was provided at no cost [32]. Failure to accept patients' reasons for nonadherence may contribute to the negative attitudes sometimes expressed by providers towards defaulting patients, resulting in difficulties in patients returning to treatment following missed appointments.

Conflicts between treatment and work and the hidden costs of treatment, resulting in expenses exceeding resources [22,26–28,31,32,34,42,43,48,54,55], could push people into poverty. This possibility was cited both by health professionals and by patients as a reason for nonadherence [23,26,32,37,42,54–56]. Males (as head of households and often sole wage earners) tended to cite this reason more frequently than females [26,37,42,55]. In societies where female or adolescent patients depend on family for financial support (particularly India and Pakistan), poverty was reported as a major reason for nonadherence to treatment [22,23,36,51,55]. For patients living in poverty, the quality of food consumed while on TB treatment was reported to affect adherence [22,26,27,29,37,45,54]. Patients reported not being able to take medication on an empty stomach, or being unable to remain in hospital due to a lack of free food [26,29,37,45,54].

### Knowledge, Attitudes, and Beliefs about TB Treatment

Many studies centred on the influence of patients' understanding of treatment, including its duration and the consequences of defaulting, on adherence to treatment [23,24,26–28,33,34,36,38–40,42,44,46,52,57]. The long treatment period was poorly understood by patients [23,26,28,38–40,46,52]; and adherence appeared to be facilitated where patients understood the importance of completing treatment [24,26,32,36,39,44,55,58,59]. One study on adherence to prophylaxis reported that nonadherent patients had little information on TB as a disease, but were very aware of the potential adverse effects caused by treatment [44].

Patients' beliefs about the efficacy of treatment, both positive [39,41,52,59] and negative [22,23,26,28,32,34,36,39,44,52,54–56], may impact on adherence. Patients may question the efficacy of the pills or think that only injections are “medicine” [22], or even question the validity of diagnostic tests that are not considered sophisticated enough for such a dangerous disease [52]. Belief in treatment efficacy appeared to be related to patient confidence in the medical system [25,35,42]; in some cases community-based treatment programmes increased confidence among community members that TB could be cured [37,55]. Another study noted that patients preferred to consult traditional healers [34].

Fear and denial of diagnosis were common themes across the included studies. Some patients had difficulty accepting their diagnosis, often wanting to hide their disease [23,29,33,40,42,43,55,56]. In other studies, patients' desire to be cured was cited as a motivator for adherence in people presenting with TB symptoms [30,41,43,46,58,59], and patients' fear of the negative consequences of irregular treatment was associated with treatment adherence [30,32,39,54].

Patients could be nonadherent if they were taking other western [46] or traditional [51,52] medicines and perceived there to be negative consequences if these were taken concurrently with TB medication. Two studies mentioned a relationship between pregnancy and nonadherence [54,55], one of which noted that female patients believed that pregnancy would increase intolerance to drugs and make TB drugs ineffective.

### Law and Immigration

In studies with IDUs and homeless people, mainly conducted in the US, legal and immigration requirements had an important influence on whether people adhered to prophylactic regimens. For refugees entering the US with inactive TB, obtaining certification of preventive treatment completion was a motivator for returning to the clinic [53]. Others also on preventive treatment were concerned that TB would affect their immigration status [60], that their illegal residence status would be discovered when accessing treatment [61], or that they would be incarcerated [62]. Some patients simply stated that they adhered because it was legally required [59]. In The Gambia, nonadherence was attributed by staff to Senegalese patients coming to the country for free treatment and returning home when feeling better [27].

### Personal Characteristics and Adherence Behaviour

Patients and providers thought that an individual's personal character determined whether they would adhere to treatment or not [24,25,28,36–38,49,57,63]. Substance abuse was noted frequently as a barrier [24,25,28,36–38,49,57,63]. Patients with mental illness [49,57]; particular ethnic groups, such as Hispanic patients in the US [49]; older and younger age groups [42,49]; and those who were residentially mobile [25,27,49,62] were considered to be at “high risk” for nonadherence by providers and patients. Religion [30,49] and personal motivation [22,27,37,39,46,54,57] were regarded as important influences on TB treatment adherence. Female patients were perceived as being more motivated [38,57], but in some countries they required permission from men or heads of household to attend treatment [27,51]. Two studies indicated that female patients who were, or wanted to be, pregnant were less likely to adhere to treatment as they perceived the medication to be harmful [54,57].

Some providers expressed the opinion that difficulties with adherence lay almost entirely with the patients [46], and used labels such as “difficult cases” for nonadherent patients [24,27,38,53]. Nonadherent patients were judged to lack interest [39], to be lazy and not care [53], or to want to remain sick to qualify for financial support [41]. Patients were criticised for not actively seeking treatment [26,29], and in one case patient characteristics were used to identify and exclude from treatment those considered at higher risk for nonadherence [25]. Wealthier, more educated people were deemed more likely to adhere [29], and illiterate patients more likely to default [22]. Two studies noted that a structured environment away from home could facilitate adherence [28,57]. Studies involving people living with HIV/AIDS noted the relationship between adherence and coping psychologically with their HIV diagnosis [64,65].

Personal agency was an important aspect of adherence behaviour; self-administering patients [22] and those who developed their own reminders adhered readily [54]. It appeared to be easier for male than female patients to be in control of the treatment process, but in one study patients felt the DOT system had transformed them from an adult to a minor, because it prevented them from managing their own treatment [42].

### Treatment Side Effects and Adherence

The influence of side effects—real, anticipated, or culturally interpreted—on adherence to treatment was mentioned in a number of studies [24,32,34,38,39,46,53,54,58]. Some patients reported stopping medication because of adverse effects [44,46] while others reported that they were not informed about side effects and what to do to counter them [25,34,58]. In some cases, patients had not communicated side effects to providers [38]; in others, the health care worker had not given attention to the side effects that patients reported [24,32,36], or had responded derisively to the patient's attempt to enquire about them [24]. Few patients acknowledged that side effects had influenced their decision to abandon treatment [51,54]. Cultural interpretations of side effects varied. For example, Vietnamese refugees with inactive TB interpreted treatment side effects as “hot” or “non-hot” and countered these effects differently [36].

### Family, Community, and Household Influences

A main theme across the included studies was the influence of community members or peers on treatment-taking behaviour [33,53,58], and the strong influence of stigma among family and friends [22,26–28,34,36,40,42,46,52,55,56,58,59,61,64]. TB patients may hide their diagnosis [26,27,29,34,37,38,40,42,56], and feel guilt and shame because of the disease [26,31,33,34,42,52]. Stigma may also make patients afraid to ask for support from their employer to purchase medication, thereby reducing adherence [29,65].

Sometimes a patient's role and responsibilities in the family could motivate them to adhere to treatment in order to recover and resume those duties [22,40,43,58,64,65]. But responsibilities in the home, such as providing income and caring for children, also reduced the likelihood of adherence for some [32].

Family support, including financial assistance, collecting medication, and emotional support, appeared to be a strong influence on patient adherence to treatment [22,26,27,29,34,36,38,40,42,52,55,56,58,59,61,64]. In some cases patients on treatment became increasingly demoralised and more likely to become nonadherent as family support weakened [23]. Providers in a study in Vietnam noted that support for the patients seemed to exist only in the family [29]. Having family members observe treatment taking was considered important for some patients, especially if the observer was a decision maker in the family [53], or a respected family member [48]. Husbands and other males' support was considered important for female patients [53]. Providers in one study noted that patients also could support each other through their treatment course [45].

Several studies reported that TB status could affect marriage [22,27,34,36,42,44,55,56]. In some cultures, females diagnosed with TB are at risk of divorce, of their husband taking a second wife, or of being sent to their natal homes [27,36,43,55]. In South Africa, red urine (a side effect of medication) was interpreted as harmful to the partner, causing abstinence from sex and thus familial disharmony and consequently potential nonadherence [34]. In Pakistan, parents' perceptions of marriage prospects influence treatment taking or avoidance among unmarried children [22,43,55].

## Discussion

The themes identified in this interpretive review were intricately linked and likely to have a combined effect on patient adherence to TB treatment. Secondary interpretations (by authors of included papers) allude to the complex, dynamic nature of adherence to TB treatment. One author suggested that patients experienced three layers of barriers to adherence: attending the health care facility initially, attending repeatedly, and experiences while there [31]. The layers were considered to be interlinked and exacerbated by geographic, economic, and gender inequalities; and patient decisions in relation to treatment taking were thought likely to shift for various reasons during the treatment course. Other authors considered adherence a chain of responsibilities including patients' behaviour, health care workers' conduct, and decision makers' and society's outlook [58]. These secondary (author) interpretations influenced our approach towards a higher-order interpretation (third-order interpretation), which distilled the translations into a whole, more complete interpretation. Based on the translated themes and secondary interpretations, we developed a model to depict our understanding of the main influences on adherence (Figure 3). Components of the model include structural, personal, and health service factors influencing adherence, as well as social context. We have presented structural factors and health service factors separately, instead of as a single “health systems” category, because we felt that some interventions could be directed towards wider society-level factors while others could intend to influence the person and the health care service.

### Structural Factors: Poverty, Gender, and Discrimination

Structural factors are those factors present in society that influence treatment-taking behaviour, but over which a patient has little personal control. Structural factors have been defined as barriers or facilitators that relate to economic, social, policy, organisational, or other aspects of the environment [66]. Factors such as gender and poverty determine individual responses to treatment and subsequent behaviour; and they interact with a patient's social context, their personal characteristics, and the health care service. TB programme managers frequently assume that a willingness to adhere must be instilled in patients in order to improve adherence rates. Our synthesis has found that even where patients are willing to adhere, structural factors such as poverty and gender discrimination may prevent them from doing so. It is recognised that incorporating patients' views in medical practice often obscures the real constraints on agency that some patients experience [9]. In our synthesis, structural factors were discussed in various ways, with poverty remaining one of the most important of these for treatment taking, especially when linked to health care service factors, such as poorly accessible, poorly equipped, and distant clinics. Our findings support the assertion that interventions to increase adherence should focus not only on the patient but also on the wider context and the health care system [67]. There is a need for a shift in perspective to give greater attention to both the social and economic environment in relation to TB infection, of which the beginnings can already be seen in the international policy arena [68].

### Patient Factors: Motivation, Knowledge, Beliefs, and Attitudes and Interpretations of Illness and Wellness

Patient choice in taking treatment is framed by the physiological and psychological impacts of the disease and also by the social and cultural structures in which the person is immersed [68]. Patient motivation and willingness, and the effect of incentives on treatment taking, have received some attention [69]. However, it remains unclear whether the incentive, or the attention received by the patient, serves as the primary source of motivation [67]. Caution should therefore be exercised when attributing adherence solely to “personal motivation” [22,27,37,39,46,54,57], because not only can important influences be ignored, but this factor is difficult to modify or even operationalise.

We found that personal and social factors, including poverty and social marginalisation, may be used by some providers to identify patients at risk of nonadherence to their medication regimen. However, it cannot be assumed that all individuals sharing a particular characteristic face the same barriers to adherence. Nonadherence can be a product of programme failures, such as an inadequate supply of drugs, rather than patient-related problems or failures [24]. Our synthesis also found that patient knowledge, attitudes, and beliefs about the disease TB, TB treatment, and patient interpretations of illness and wellness, can act as a “filter” for the information and treatment offered by the health services. The influence of patients' interpretation of various illnesses on their adherence behaviour is well documented, and it is recognised that patients may interpret the themes of illness, wellness, and disease differently from health professionals [70–73], highlighting the distinctions between lay and biomedical understandings of TB [10]. This is unlikely to be the only influence on treatment taking, however, and patient interpretations can interact with structural and health care service factors as well as with social context.

### Social Context

The influence of social context on treatment adherence was apparent in all included studies. The community, household, and health care service helped in countering the shame and guilt that patients with TB experienced, and also offered support in maintaining treatment taking. Social support can help patients overcome structural and personal barriers, and may influence their knowledge, attitudes, and beliefs. Conversely, community and family members' attitudes may influence a patient's decision to stop taking TB treatment. In such circumstances, community-based TB treatment programmes and stronger involvement of local social networks to support TB patients may be justified [6].

### Health Care Service Factors

Factors related to the provision of health care services emerged strongly in the synthesis. Flexibility and choice in treatment, and options that maintain patient autonomy in treatment taking, appeared to run contrary to the traditional organisation of many TB services [6,10]. These problems were exacerbated by programme failures, such as inadequate supplies of drugs [23,33,36,38] and difficulties in consulting providers [22,23,31,36–38]. DOT at a health care facility often meant that a patient had to give up part of their working day to attend [22,23]. However, responsibilities in the home, including providing for their family, may be given priority over treatment adherence by patients. Other health care service factors, such as long waiting times and inconvenient opening times in clinics, add to economic discomfort and social disruption for patients [49], and negatively influence adherence. The studies suggest that patients often face a choice between employment and taking medication for TB; and there is evidence that patients consciously estimate the opportunity costs of taking treatment.

### Study Limitations

The majority of studies included in this synthesis were conducted in developing countries; the findings are therefore most applicable to low- and middle-income countries that carry the greatest burden of TB disease and where interventions to improve treatment completion are needed urgently. The findings may also be applicable to countries with better resources; indeed, a meta-ethnography of treatment taking in high-income countries showed findings similar in many ways to those of our study [74]. The clustering of studies by region may have been due to the difficulties of locating primary studies, and may have produced some of the similarities between issues described by participants.

Studies often included participants from several socioeconomic strata; did not always contain a detailed description of the treatment regimen; and did not explicitly consider gender in treatment adherence. Therefore it was not always possible to tease out similarities or differences in the identified themes based on these characteristics. We identified some patterns relating to the type of treatment intervention—for example, direct observation versus patient-administered treatment—but the majority of studies did not describe adequately interventions or treatment regimens. Our observations regarding gender differences in taking TB treatment are dependent largely on the information provided by original authors. Collecting author (secondary) interpretations proved difficult; most authors maintained a descriptive style in presenting their findings and so the distinction between findings and interpretation was often not clear.

It is important to consider the effect on the review findings of combining studies from different theoretical traditions, and this is widely debated. We found that the level of interpretation in the included studies was fairly basic—most were descriptive studies that used thematic analysis to identify key themes and did not draw extensively on theory or on a particular theoretical tradition. While this made it more feasible to combine the study findings, it also meant we were unable to explore any differences in interpretation of factors affecting adherence in studies conducted within different theoretical frameworks.

### Implications for Policy and Practice

Using the reconceptualised model of factors influencing adherence to TB treatment (Figure 3), we consider it important that policy makers, practitioners, and patient support groups acknowledge: patient autonomy in the treatment process; the importance of patient-centred interventions that encourage shared decision-making regarding treatment; the role of support systems tailored to patient needs; the role of informal, societal structures in reinforcing adherence through patient support; and the influence of poverty and gender on patients and their treatment adherence.

New interventions to promote treatment adherence could be designed with these factors in mind. For example, when known barriers to adherence are mapped against the currently available interventions to promote adherence, it is interesting to note that very few interventions are designed to build on social and family support mechanisms. Most are targeted at overcoming barriers to health care delivery to the individual [75].

Based on our third-order interpretation, we identified a number of hypotheses that may guide policy makers and practitioners in developing and implementing specific measures to improve adherence, including influencing the behaviour of practitioners, the organisation of services, and the behaviour of individuals (Box 9). This review shows the usefulness of qualitative synthesis in informing policies for health interventions. Through bringing together data from multiple primary studies, and looking for commonalities across these studies, the approach provides fresh insights into the reasons for poor adherence and guidance on where the development of more patient-centred interventions to improve adherence could be useful. Such insights can be useful to both programme managers at local and national levels and also in facilitating the development of more appropriate international policies for the management of TB.

**Box 9. Factors Likely to Improve TB Treatment Adherence**

- Increase the visibility of TB programmes in the community, which may increase knowledge and improve attitudes towards TB
- Provide more information about the disease and treatment to patients and communities
- Increase support from family, peers, and social networks
- Minimize costs and unpleasantness related to clinic visits and increase flexibility and patient autonomy
- Increase flexibility in terms of patient choice of treatment plan and type of support
- Increase the patient centredness of interactions between providers and clients
- Address “structural” and “personal” factors, for example through micro-financing and other empowerment initiatives
- Provide more information about the effects of medication to reduce the risk of patients becoming nonadherent when experiencing treatment side effects

### Implications for Research

Based on the findings of this synthesis we believe that further research is needed both to understand people's experience of TB and its treatment and to develop more patient-centred approaches to improving treatment adherence among people with TB. By “patient-centred approaches” we mean interventions that focus on sharing decisions about interventions or the management of health problems with patients and that view the patient as a whole person who has individual preferences situated within a wider social context [76].

Key issues to be explored in this research include how gender shapes experiences of treatment taking and how differing gender roles may influence adherence. This aspect was reported less frequently than expected in the primary studies in this review and would benefit from further exploration. Patient experiences of side effects of treatment, and how these influence decisions to stop taking treatment, also warrant further research since the existing literature reports vary as to the influence of side effects on treatment adherence [77,78].

There is also little published evidence on the experiences of patients living with HIV/AIDS and taking treatment for TB or receiving concurrent treatment for both diseases; our review included only three reports of qualitative research in this area [51,63,64]. The small number of studies is surprising, given the high rates of TB–HIV coinfection, especially in sub-Saharan Africa [79]; the complex treatment regimens involved; and the need for high rates of treatment adherence for both diseases. There is also some evidence that where coinfection is common, a diagnosis of TB may be seen as a diagnosis of HIV and this “form” of TB may be seen as incurable, with consequent impacts on patient adherence to treatment [80]. Managing treatment for both HIV and TB is therefore likely to present unique challenges to patients, providers, and the health care system, and further research on the particular experiences of patients taking antiretroviral and anti-TB treatment would be very helpful.

The process of data extraction and quality assessment identified a number of lacunae in the included study reports. Studies frequently failed to report the details of how treatment was delivered, for example whether direct observation of treatment was used; the treatment regimens used; and the sociodemographics of the included study populations. Greater attention to these areas would improve understanding of research findings and facilitate assessment of their transferability to other contexts. The reporting of a number of study quality issues also needs to be addressed in future reports, including the theoretical orientation of the research and sampling and analysis approaches (see Table 1).

Finally, lay conceptualisations of illness and wellness, particularly of TB and its treatment, are not well understood. The TB treatment literature is almost entirely conceptualised from a biomedical perspective, and even studies of patient experiences are largely conducted with the aim of improving treatment adherence. Understanding lay conceptualisations will help in comprehending why people may stop taking treatment at particular times. This would involve acknowledging that patients have agency and are active [71] in shaping their own treatment decisions rather than seeing poor adherence simply as “irresponsible” behaviour. Research approaching TB adherence from a nonbiomedical perspective is required to further understand the impact of traditional beliefs [81] and perceptions of illness and wellness on adherence to treatment. Any further work on patient experiences of TB adherence should also acknowledge and explore the social, economic, and geographical contexts in which a patient is located.

There are suggestions that the growing interest in the subjective experiences of health care consumers may result in these experiences being used as simply another tool with which to better promote treatment adherence. In addition, this focus, and its attendant notions of shared responsibility for treatment between consumers and providers, could be seen as acting to expand the surveillance of treatment taking from health care workers to consumers and the wider community [82,83]. We therefore believe it is important that this kind of evidence is used carefully by decision makers and practitioners. The extent to which new interventions come from biomedical rather than lay perspectives should be recognised to ensure that structural factors, as well as individual patient responsibilities in treatment taking, are considered.

### Conclusion

This synthesis indicates that patients often take their TB medication under difficult circumstances and experience significant challenges, many of which are outside of their direct control. Taking a lengthy course of medication is not straightforward and frequently involves difficult decisions, sometimes at substantial personal and social cost to the patient. Adherence is a complex, dynamic phenomenon; a wide range of interacting factors impact on treatment-taking behaviour, and patient behaviour may change during the course of treatment. More patient-centred interventions, and far greater attention to structural barriers, are needed to improve treatment adherence and reduce the global disease burden attributable to TB.

## Supporting Information

Alternative Language Abstract S1 Translation of the abstract into Norwegian by Atle Fretheim (48 KB PDF)

Table S1 Search Results (35 KB DOC)

## Abbreviations

DOT: direct observation of therapy
DOTS: directly observed treatment, short course
IDU: injecting drug user
TB: tuberculosis
