# Supplementary material for: Patient Adherence to Tuberculosis Treatment: A Systematic Review of Qualitative Research
Source: PLoS Med. 2007 Jul 24;4(7):e238. doi: 10.1371/journal.pmed.0040238 (PMC1925126; doi:10.1371/journal.pmed.0040238)
Supplement: Supplementary file 1 [file pmed.0040238.sd001.pdf]

Sammendrag

Bakgrunn

Tuberkulose står for en betydelig del av den globale sykdomsbyrden og sykdommen har fått mye oppmerksomhet de seinere årene, særlig i fattigere land hvor den assosieres med HIV/AIDS. Det er et utbredt problem at mange pasienter ikke gjennomfører behandlingen til tross for de mange tiltak som benyttes for å sikre at medikamentregimet etterleves. Det mangler en helhetlig forståelse av hvilke faktorer som er avgjørende for hvorvidt pasientene fullfører behandlingen eller ei, og dette er et viktig hinder for å finne fram til effektive løsninger. Målet med denne systematiske oversikten over kvalitative studier var å forstå hvilke faktorer som pasienter, omsorgspersoner og helsepersonell mener er viktige med tanke på gjennomføring av tuberkulosebehandling.

Metode

Vi søkte i19 elektroniske databaser (1966-februar 2005) etter kvalitative studier som omhandlet oppfatninger til pasienter, omsorgspersoner eller helsepersonell vedrørende gjennomføring av forebyggende eller kurativ tuberkulosebehandling, med fritekst ordene “Tuberculosis AND (adherence OR compliance OR concordance). Vi supplerte søket med henvisningssøk og konferering med eksperter. For studier som ble inkludert vurderte vi kvalitet ved hjelp av en forhåndsbestemt sjekkliste, og data ble uavhengig ekstrahert til et standardisert skjema. Vi sammenstilte våre funn ved hjelp av Noblit og Hares metode for metaetnografi, gjennom en iterativ fortolkning av de enkelte studiene mot hverandre og en argumentasjonslinjesyntese.

Funn

Vi gjennomgikk 7814 titler og valgte ut 44 artikler som oppfylte våre forhåndsdefinerte inklusjonskriterier. Sammenstillingen gir en oversikt over kvalitativ kunnskap utledet fra alle disse internasjonale studiene. Vi identifiserte åtte hovedtema på tvers av studiene: den økonomiske byrden ved tuberkulosebehandling; organisering av behandling og omsorg; kunnskap, holdninger og overbevisninger med tanke på behandling; fortolkninger av sykdom velvære; støtte fra familien, nærmiljøet og husholdningen; personlige faktorer; bivirkninger; og lovgivning og migrasjon. Vår fortolkning av temaene på tvers av alle studiene ledet fram til en syntese i form av en argumentasjonslinje som viser hvordan fire hovedfaktorer virker sammen og påvirker hvorvidt behandlingen blir gjennomført: strukturelle faktorer, som fattigdom og kjønnsdiskriminering; sosiale rammer; faktorer knyttet til helsetjenesten; og personlige faktorer.

Konklusjoner

Pasientens beslutning om å avslutte behandlingen var et resultat av en rekke samvirkende faktorer. Etterlevelse av den langvarige tuberkulosebehandlingen er et kompleks, dynamisk fenomen hvor et vidt spekter av samvirkende faktorer spiller inn på pasientens atferd. Våre funn kan bidra til utviklingen av pasientrettede tiltak, og av tiltak rettet mot strukturelle barrierer som hindrer at pasienter fullfører behandlingen.
