# Supplementary material for: Patient Adherence to Tuberculosis Treatment: A Systematic Review of Qualitative Research
Source: PLoS Med. 2007 Jul 24;4(7):e238. doi: 10.1371/journal.pmed.0040238 (PMC1925126; doi:10.1371/journal.pmed.0040238)
Supplement: Supplementary file 2 [file pmed.0040238.st001.doc]

# Table 1: Outputs from database searches*

| **Database** | **Years searched** | **Total number of citations identified** |
| --- | --- | --- |
| PsycINFO | 1972- | 53 |
| ERIC | 1966- | 6 |
| Academic Search Premier | 1965- | 205 |
| Health Source: Nursing/Academic | 1985- | 141 |
| ScienceDirect | 1964- | 149 |
| Social Science full text | 1983- | 29 |
| Social science citation expanded, social science citation index, arts and humanities citation index | 1975- | 889 |
| Medline | 1966- | 1772 |
| CINAHL | 1982- | 321 |
| Pre-CINAHL | Current | 1 |
| Dissertation abstracts | 1980- | 57 |
| Sociological abstracts, social services abstracts, PAIS international | 1963-, 1972-, 1980- | 17 |
| EMBASE | 1966- | 2349 |
| PapersFirst | 1993- | 12 |
| Pubmed | 1966- | 1813 |
| Reference/ recommendation | - | 1 |
| **Total number of citations** |  | 7815 |

* All databases searched from the dates above to February 2004. Additional articles were included as these became available. Total number of citations includes duplicates.
